# Wearable Sensor-Based Rehabilitation Exercise Assessment for Knee Osteoarthritis

**DOI:** 10.3390/s150204193

**Published:** 2015-02-12

**Authors:** Kun-Hui Chen, Po-Chao Chen, Kai-Chun Liu, Chia-Tai Chan

**Affiliations:** 1 Department of Biomedical *Engineering*, National Yang-Ming University, 155, Li-Nong St., Sec.2, Peitou, Taipei 11221, Taiwan; E-Mails: orthochen@gmail.com (K-.H.C.); gfd9885@gmail.com (P.-C.C.); g30104026@ym.edu.tw (K.-C.L.); 2 Department of Orthopaedic Surgery, Taichung Veterans General Hospital, 1650 Taiwan Boulevard Sect. 4, Taichung 40705, Taiwan

**Keywords:** knee joint rehabilitation exercise, wearable sensor, rehabilitation assessment system

## Abstract

Since the knee joint bears the full weight load of the human body and the highest pressure loads while providing flexible movement, it is the body part most vulnerable and susceptible to osteoarthritis. In exercise therapy, the early rehabilitation stages last for approximately six weeks, during which the patient works with the physical therapist several times each week. The patient is afterwards given instructions for continuing rehabilitation exercise by him/herself at home. This study develops a rehabilitation exercise assessment mechanism using three wearable sensors mounted on the chest, thigh and shank of the working leg in order to enable the patients with knee osteoarthritis to manage their own rehabilitation progress. In this work, time-domain, frequency-domain features and angle information of the motion sensor signals are used to classify the exercise type and identify whether their postures are proper or not. Three types of rehabilitation exercise commonly prescribed to knee osteoarthritis patients are: Short-Arc Exercise, Straight Leg Raise, and Quadriceps Strengthening Mini-squats. After ten subjects performed the three kinds of rehabilitation activities, three validation techniques including 10-fold cross-validation, within subject cross validation, and leave-one-subject cross validation are utilized to confirm the proposed mechanism. The overall recognition accuracy for exercise type classification is 97.29% and for exercise posture identification it is 88.26%. The experimental results demonstrate the feasibility of the proposed mechanism which can help patients perform rehabilitation movements and progress effectively. Moreover, the proposed mechanism is able to detect multiple errors at once, fulfilling the requirements for rehabilitation assessment.

## 1. Introduction

Since the knee joint bears the full weight of the human body and the highest pressure loads while providing flexible movement, it is the body part most vulnerable and susceptible to osteoarthritis (OA) [1]. According to the annual medical census in 2010 published by the Taiwan Department of Health, the number of patients with arthropathies and related disorders is about 14% of the total number of outpatients and inpatients in Taiwan; and 80% of patients with knee OA are above 50 years old. Furthermore, the number of people above the age of 50 makes up 30.6% of the total Taiwanese population in 2011, and this ratio will become higher in the future. In the United States, approximately 9.29% of the US population is diagnosed with symptomatic knee OA by the age of 60. According to the 2012 census of the U.S. Department of Commerce [2], the number of U.S. residents above the age of 50 made up 32.08% of the total resident population in 2010. Therefore, the study of issues related to knee OA are becoming more important for both the current and the future society.

Treatments for knee OA include medication, exercise therapy and surgery. Above all, exercise therapy is an important interventional treatment for mild to moderate stages of knee OA [3]. Although exercise therapy may improve symptoms, it does not reduce the knee adduction moment, a key indicator of OA disease progression [4]. Patients with knee OA receive exercise therapy through a series of rehabilitation programs that are beneficial to joint mobility and body metabolism. The early rehabilitation stages last for approximately six weeks, during which the patient works with the physical therapist several times a week. The patient is afterwards given instructions for continuing exercise at home [5]. An appropriate rehabilitation exercise can relax joint capsules and ligaments, prevent osteoporosis, strengthen muscles around the knee and increase active weight-bearing ability. However, improper rehabilitation exercises not only put patients on risk of slower recovery, but also may cause more damage by adding stress to the injured parts of the knee. Therefore, developing a rehabilitation exercise assessment system that monitors patients' quality and accuracy of rehabilitation movements at home can play an important role in the success of a patient's recovery process. Recently, a tri-axial accelerometer was widely adapted to recognize daily human activities in several well-known research projects [5–7]. In the research published by Taylor [5], the authors used a peak detection method to divide signals into time segments, and then extracted features of both the frequency and time domain from each time segment to detect improper movements through a classification algorithm. During the first set of each exercise, the subject performed the exercise as interpreted from the instructions. The video was captured for data of offline labeling. The expert was asked to score the labels for exercises by occurrence in real world observation and by severity of the error. The improper movement labels were defined by both high occurrence and severity. A non-expert labeled the remaining data using guidance from the expert. Their system was applied in the three knee OA rehabilitation exercises, and was able to let their users know how to modify their movements. However, their system was unable to detect multiple errors at once, and the accuracy was affected by labeling inconsistency.

In our work, three wearable sensors, tri-axial accelerometers, are mounted on the chest, thigh and shank of the working leg. The proposed mechanism uses the angle information and both the time-domain and frequency-domain features of the sensor signals to identify the type of rehabilitation exercise by a decision tree and detects improper exercise movement by hierarchical methods. The rehabilitation exercise assessment system for knee OA can help patients self-manage their rehabilitation progress at home, and when the improper exercise posture is detected, an error alarm can be provided for the patients to verify their movements in real-time. Moreover, during the follow-ups at outpatient clinics or medical departments, the physicians can assess the patients' progress and how effectively the rehabilitation activities were carried out through the recording of the system. The rest of this paper is organized as follows: Section 2 introduces a literature review of rehabilitation assessment systems. The materials and methods are described in Section 3. Section 4 presents the experimental results; Section 5 concludes our work.

## 2. Related Work

Over the past decade, several well-known research projects have studied physical activity recognition [6,7]. The methods for physical activity recognition can be used in body movement classification, which can help users manage their exercise progress, and also be used to assess the effectiveness of rehabilitation and physical functional performance.

### 2.1. Physical Activity Recognition

Home telecare services are a trend of the future because of the aging population and limited funding for public health care. The ability to record and classify humans' motions are essential when attempting to assess their functional ability and general level of activity. Karantonis *et al.* [8] proposed a system that could classify in real-time the types of human movement associated with data acquired from a single tri-axial accelerometer unit worn at the hip. The major function of their system was discriminating between periods of activity and rest, recognizing the orientation of the posture, detecting events such as walking and falls to a reasonable degree, and estimating of metabolic energy expenditure. Ermes *et al.* [9] used Global Positioning System (GPS) and information from tri-axial accelerometers mounted on the waist and hip to recognize daily activity. Their study drew a comparison between unsupervised settings and supervised settings, focusing on how well the subjects' daily activities could be recognized. Yang *et al.* [10] used the signal of a tri-axial accelerometer worn on the lower back of the subjects to assess whether the subjects were victims of complex regional pain syndrome. The proposed classification mechanism extracted the discriminative features and used artificial neural network to recognize gait patterns. The proposed system could provide objective assessments of patients' physical functional performance to evaluate the outcome of therapy.

Miniature body-worn sensors are suitable for collecting data on activity patterns over extended periods of time. Preece *et al.* [7] reviewed the different techniques which have been used for activity recognition or fall detection using body-mounted sensor data. There are four steps to approach activity recognition, which include sensor signal acquisition, windowing techniques, feature generation and classifying algorithms. Wearable sensors include accelerometers, gyroscopes, magnetometers, *etc.* Accelerometers respond to gravity as well as to their acceleration, and it can be used to estimate the inclination of a body segment, body sway, or for measuring activity levels, however accelerometers have some limitations such as noisy signals and difficulties in estimating the gravity vector accurately during dynamic movements because the movement itself also contributes to the acceleration measurement, not just gravity. A gyroscope is sensitive to electronic noise and temperature effects [11], and it suffers from increased errors while integrating signals to estimate orientation information. Some researchers have focused on sensor fusion to combine and leverage the strengths of different sensors together, e.g., accelerometers and gyroscopes [12]. For windowing techniques, there are three different approaches: sliding window, event-defined window and activity defined window. The sliding window divides the signal into segments of fixed length, and it does not require preprocessing of the sensor signals, which is well suited for real-time applications. With event-defined windows, pre-processing is required to locate specific events, such as heel strike or toe-off. The size of each window is not fixed. Activity defined windows divide time segments depending on determining the times at which the activity changes. Feature generation methods contain heuristic, time domain, frequency domain and time-frequency features. There are many algorithms for classification techniques, such as hierarchical methods, Bayesian classifier, *K*-nearest neighbor, decision tree, *etc.*

Hierarchical methods are similar to binary decision trees. A binary decision is made at each node based on the input features. Each node maybe generates a definite result or is a transition to another node, where a further decision is made. The decisions made at each node are based on manual inspection and analysis of the training data. Fahrenberg *et al.* [13,14] used a hierarchical approach to classify four activities. They extracted time domain features from accelerometers mounted on the chest, wrist, shank and thigh [15]. However, they were unable to discriminate between level walking and stair walking in the between-subject design. The Bayesian classifier is a statistical approach based on the estimated conditional probabilities or likelihoods of the signal features available from each class. Given such likelihood, the Bayesian approach can estimate the probability of a new unknown pattern having been generated by a specific activity. With the assumption of features independent with each other, the likelihood function for each activity can be expressed as the product of *n* simple probability density function, where *n* is the number of features. Further details can be found in the work of Duda *et al.* [15] and Theodoridis *et al.* [16–18]. To implement K-nearest neighbor, a multi-dimensional feature space will be constructed. Each dimension corresponds to a different feature. Firstly, all training data points locate within the feature space, where each point represents a specific activity. A testing data point will be classified by the majority of the K-nearest points of identified training data which correspond to a given activity. The value of K typically ranges from 1 to a small percentage of the training data and is selected using trial and error. Foerster *et al.* [19] were the first one to use K-nearest neighbor for activity recognition. They extracted time domain features from four accelerometers mounted on the sternum, wrist, thigh and lower leg. Their system was able to classify between nine common activities (sitting, standing, lying supine, sitting and talking, sitting and operating a PC keyboard, walking, stairs up, stairs down and cycling) using a within-subject design. In their subsequent work [20], the proposed method used the time domain and frequency domain features to improve activity classification. It combined a K-nearest neighbor classifier and hierarchical decision structure as classification method to discriminate a wider range of activities. The decision tree method is similar to the hierarchical method. In comparison with hierarchical models constructed manually by the user, decision trees can automate the process and create a complex set of rules. The decision tree algorithm works by examining the discriminatory ability of the features one at a time to create a set of rules which finally leads to a complete classification structure. Further details can be found in Godfrey *et al.* [21], Quinlan [22] and Bao and Intille [23].

### 2.2. Rehabilitation and Physical Functional Performance Assessment

Wearable sensing and feedback can be used for a variety of other clinical applications such as identifying movement disorders, assessing surgical outcomes, improving walking stability, and reducing joint loading [24]. The rehabilitation process of stroke patients is guided by clinical assessments of motor abilities. Best therapy selection for stroke patients is based on accurate assessment of motor abilities. The accuracy of observational assessments may vary greatly across clinicians. Wearable sensors can be used to assess motor ability more accurately or can be used in addition to observational clinical score. Hester *et al.* [25] proposed the use of a wearable sensor system to assess the motor ability of stroke patients. The proposed system used features extracted from each time segments for linear regression to predict clinical scores of motor abilities. Through this system, users could understand their progress of recovery while they were doing rehabilitation exercises at home.

Various rehabilitation exercises and protocols are used in a rehabilitation process for improving patients' health status. However, the patients should repeatedly visit clinicians in a rehabilitation center during the rehabilitation process. Brutovsky *et al.* [26] proposed a system which can let users perform the rehabilitation program at home, and provided real-time biofeedback during the rehabilitation process and informing patient about achieved results and further goals to succeed. Users can download appropriate rehabilitation protocols according to their own conditions, thus enabling this system to be used in a wide spectrum of scenarios. The system consisted of sensorial unit, visualization and communication unit and virtual clinical revision server. Sensor unit consisted of 3D acceleration sensor, microprocessor and Bluetooth module. It calculated the tilt angle of rehabilitation movement and sent it to visualization unit. The visualization unit, which was a Personal Digital Assistant (PDA), works as a guideline of rehabilitation exercises providing biofeedback. Finally, progress report was shown at the end of each exercise recapitulating achieved results in comparison to the past performance history, which allowed clinicians to monitor patients' rehabilitation progress through telecommunication on PDA.

Tseng *et al.* [27] demonstrated a system that used a tri-axial accelerometer and compass to capture human motion for home rehabilitation. The system consisted of a wireless sensing platform, a motion analysis module, and a game interface, which helped patients to conduct their rehabilitation program. The major parameters of motion were the joint angle information, angle between sensor node, and gravity direction. The game interface could motivate patients to complete the rehabilitation progress, and enabled to check if patients' performances were acceptable.

## 3. Materials and Methods

**Figure 1.**
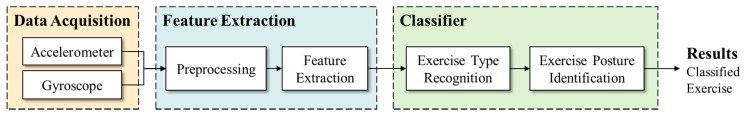
The architecture of the proposed rehabilitation exercise assessment system.

The system architecture is proposed in order to provide a reliable rehabilitation exercise assessment, as shown on Figure 1. The system consists of three main components: data acquisition, feature extraction, and classifier. The details of each are described in the following sections.

### 3.1. Data Acquisition

Several researchers have used vision-based devices such as the Kinect [28–30] and VICON [31] to approach related applications. The advantage of vision-based motion tracking devices is their accurate position information [32]. However, those devices must be placed in a fixed environment, limiting the range of users' movement. An alternative choice is a wearable sensor which allows subjects to wear it on the body and is not limited by the position and the capabilities of the camera(s). We choose OPAL produced by APDM (Portland, OR, US) as a wearable sensor to record the movements of subjects. The OPAL sensor includes an accelerometer, gyroscope, and magnetometer. This work collected data using the accelerometer and gyroscope since the magnetometer can easily suffer interference from the environment, and therefore was excluded from this study. Three OPALs are mounted on the anterior surface of the user's chest, thigh (close to the knee) and shank (close to the ankle) of the working leg, with the y-axis of the sensors well-aligned to the long axis of the body segment. The sensor on the chest recognizes the direction of the trunk movement, and the sensors on the thigh and shank recognize the leg movement parameters such as knee flexion angle and thigh raising angle. Each wearable sensor is 48.5 × 36.5 × 13.5 mm, weighs 22 grams, and has a sampling rate of 40 Hz.

**Figure 2.**
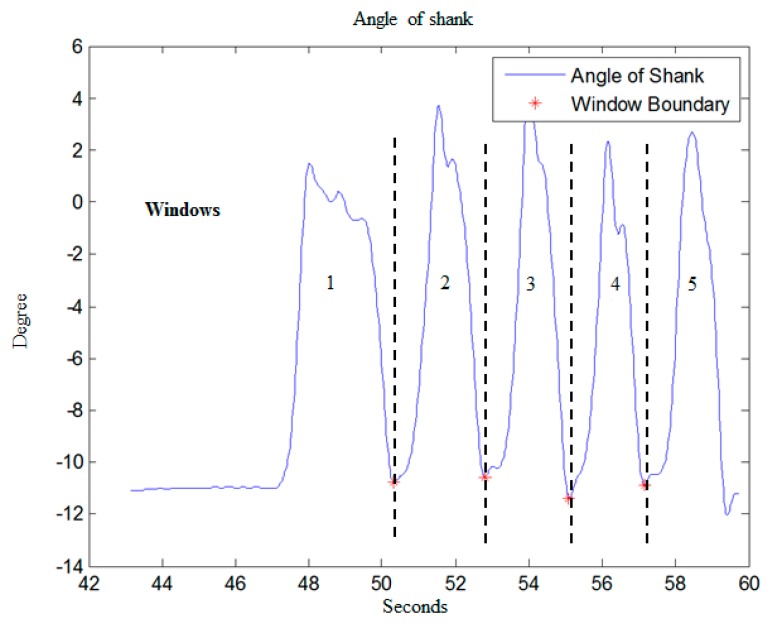
Peak detection for defining start and end point of movement.

Regarding the segmentation of time windows, the rehabilitation exercises of knee OA in this work involve periodic angle variations of the shank, so the system utilizes a peak detection method on shank angle variation to define the start and end point of every movement. Figure 2 shows the shank angle variations during SAE movement, and the red points represent signal valleys. Then time windows are divided based on the signal valley points. Through this method, each window will accurately contain each movement repetition, unaffected by the different time it takes by users to complete each movement. This will allow users to perform rehabilitation exercises at their own comfortable speed instead of a fixed time.

**Figure 3.**
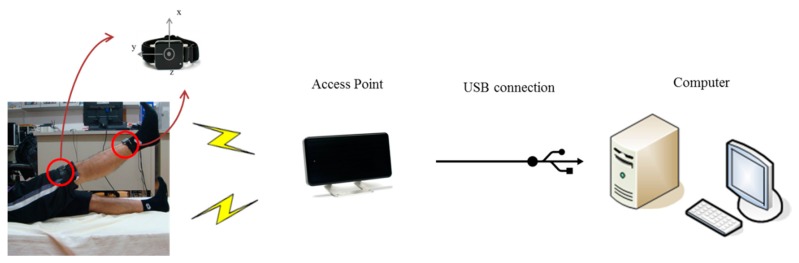
Hardware Setup.

The hardware architecture is shown in Figure 3. The access point receives signal of accelerometers wirelessly from the sensor nodes and transmits those raw data to the workstation through a USB connection. Finally, the accelerations are processed on the working station using MATLAB. The wireless transmission protocol between the accelerometers and access point is robust synchronized streaming mode, which has a typical latency of about 300–400 ms.

**Figure 4.**
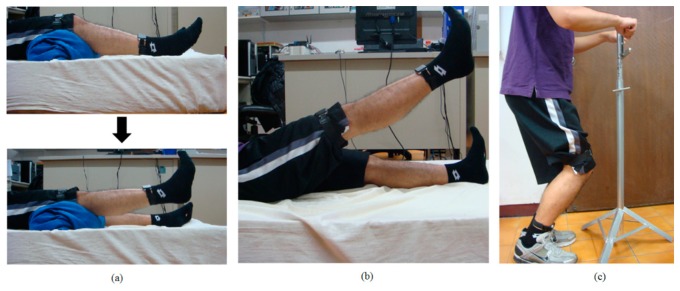
(**a**) Short-Arc Exercise (SAE); (**b**) Straight Leg Raise (SLR); (**c**) Quadriceps Strengthening Mini-squats (QSM).

For this study, data is collected from 10 healthy subjects (five males and five females, height 163.9 ± 8.9 cm, weight 56 ± 10.11 kg). The sensors placement is executed by the subject. Three types of rehabilitation exercise commonly prescribed to OA knee patients are Short-Arc Exercise (SAE), Straight Leg Raise (SLR), and Quadriceps Strengthening Mini-squats (QSM), as shown in Figure 4. These exercises are majorly in strengthening quadriceps. Strong muscle surrounding the knee can absorb impact force of weight bearing, protecting the knee joint. Moreover, it can prevent deterioration for the patients who suffered from knee pathological change [31]. SAE is a basic and important exercise in clinics, SLR is the most secure exercise for knee joint rehabilitation [31], and QSM is a weight training exercise for quadriceps, which can strengthen the muscle effectively. We label several improper alternations of those three rehabilitation exercises based on the suggestion of a physical therapist, as shown in Table 1. Each subject performs each altered movement 10 times. “Raise angle not approx. 45°” for SLR was performed 20 times because it includes both beyond and below 45°.

### 3.2. Feature Extraction

**Figure 5.**
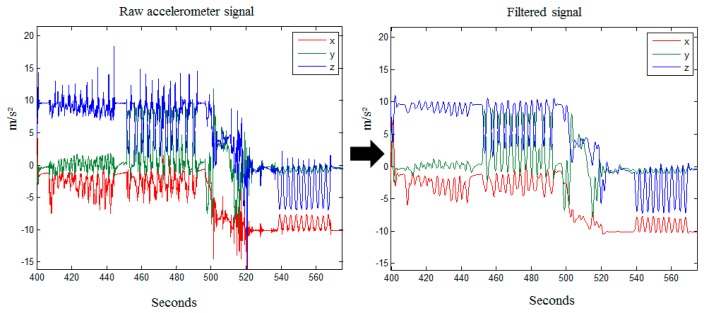
Raw accelerometer signal filtered by MF and LPF.

The system selects accelerometer and gyroscope signals as system input data to record subject movement. The input data might extract unsuitable features that may mislead the classifier and end up degrading the accuracy. Signal filters are a typically method to eliminate noise and outliers in data preprocessing [8]. This work utilizes high pass and low pass filters to preprocess the data. In the beginning the accelerations are filtered by a median filter (MF) with *n* = 3 (third order), which removes abnormal noise spikes produced by the accelerometer. Then a low pass filter (LPF) with a cut off frequency at 0.5 Hz is applied to filter the results of the median filtered signal. In the low pass filter, the gravity force components of the three axes are kept and acceleration components are filtered out, as shown in Figure 5.

**Figure 6.**
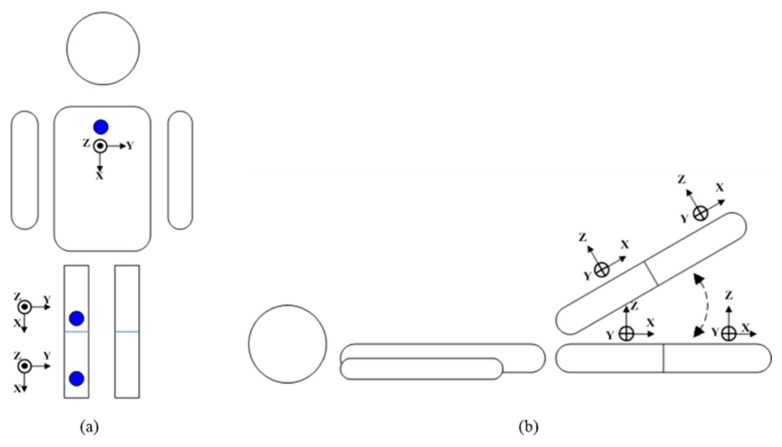
Direction of tri-axial accelerometers.

In this work, the features we use include joint range of motion, frequency-domain feature set, and mean value. There are two approaches for joint range of motion utilized in this work that in the first approach only use an accelerometer as sensor. The signals of tri-axial accelerometers are used to calculate the joint range of motion including the angle of thigh raise, knee flexion, hip external rotation and trunk forward bending to evaluate whether rehabilitation exercises will be properly carried out. The gravity force components in the three axes of the accelerometer are converted into a tilt angle. These angles can be calculated by Equations (1)–(3):

(1) $$\rho =\tan^{-1}\left(\frac{{\text{A}}_{\text{x}}}{\sqrt{{{\text{A}}_{\text{y}}}^{2}+{{\text{A}}_{\text{z}}}^{2}}}\right)$$

(2) $$\varphi =\tan^{-1}\left(\frac{{\text{A}}_{\text{y}}}{\sqrt{{{\text{A}}_{\text{x}}}^{2}+{{\text{A}}_{\text{z}}}^{2}}}\right),\text{and}$$

(3) $$\theta =\tan^{-1}\left(\frac{{\text{A}}_{\text{z}}}{\sqrt{{{\text{A}}_{\text{x}}}^{2}+{{\text{A}}_{\text{y}}}^{2}}}\right)$$

where A_x_, A_y_, and A_z_ represent gravity force components in x, y and z-axial respectively. The angles, ρ, φ, and θ, are the tilt angles between x, y, z axis and the ground. Given the example of the QSM related angles, through the calculation of the x-axial tilt angle on the chest to get the forward-bending angle of the trunk, and z-axial tilt angle on thigh to get squat angle, as shown in Figure 6a. Given another example of the SLR-related angles, the calculation of the tilt angle of x-axis to get the raise angle of the thigh and shank, tilt angle of y-axis to get the hip external rotation angle, and subtract the angle of shank from the angle of thigh to get the knee flexion angle, as shown in Figure 6b.

Another approach based on Takeda *et al.* [33] is utilized to eliminate the acceleration components and keep gravity components to calculate the inclination angles variation during movement. Gyroscope signals are utilized to estimate the rotational acceleration in each movement considering the orientation of the segments is essential for the exercise assessment. The calculations for the thigh segment can be divided into translational motion and rotational motion. Based on the accelerometer and gyroscope signal, the rotational acceleration for the thigh can be expressed as:

(4) $${\ddot{\text{r}}}_{\text{HT}}={\dot{\text{w}}}_{\text{T}}\times {\text{r}}_{\text{HT}}+{\text{w}}_{\text{T}}\times \left({\text{w}}_{\text{T}}\times {\text{r}}_{\text{HT}}\right)$$

where ṙ_HT_ is the rotational acceleration of the thigh segment, ẇ_T_ is the angular acceleration of thigh segment, r_HT_ is the distance from hip to sensor worn on thigh segment, and w_T_ is the angular velocity output of sensor worn on thigh segment.

The gravitational acceleration can be obtained by:

(5) $$\text{g}={\ddot{\text{r}}}_{\text{HT}}-{\text{O}}_{\text{T}}$$

where O_T_ is the output of acceleration sensor mounted on the thigh.

A tri-axial acceleration sensor is utilized as an inclination sensor, as it can measure the gravitational acceleration, and the output of an acceleration sensor O_i_ can be expressed as:

(6) $$O_{i}=a_{i}-g_{i}$$

where *a_i_* is the translational acceleration and *g_i_* is the gravitational acceleration. Both are measured along with the i axis of the acceleration sensor.

If the acceleration sensor is static the *a_i_* is 0, which means that the gravitational acceleration is the only output. Then, the inclination angles for the three axes of an accelerometer against the gravitational acceleration orientation can be expressed as:

(7) $$\theta_{\text{T}}=\cos^{-1}\frac{{\text{O}}_{\text{i}}}{g_{i}}$$

where **θ**_T_ is the inclination angle of thigh.

On the method of feature extraction, Preece *et al.* [7] compared time-domain, frequency-domain, and time-frequency domain features based on the same classifier and experimental environment. In order to derive frequency-domain features during exercise, the sensor data must first be transformed into the frequency domain, normally using a fast Fourier transform (FFT). The frequency-domain feature set in this work is defined as the magnitude of the first five components of the FFT power spectrum, which can get the best performance [7]. Therefore, we adopt the approach as our feature extraction method to classify exercise type.

### 3.3. Classifier

**Figure 7.**
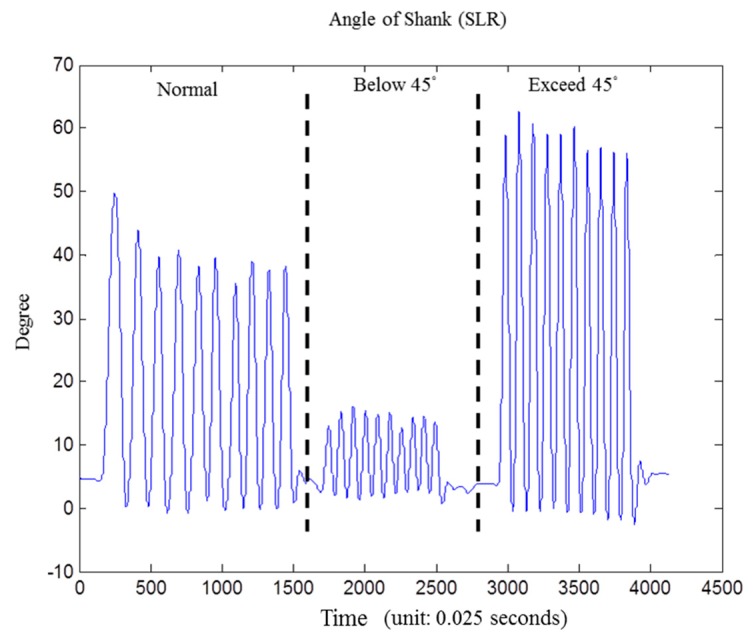
Angle information for improper movement detection of shank angle during the SLR exercise, from left to right: standard normal movement, below 45 degree, and exceed 45 degree.

In exercise classification stage, we choose decision tree for its high accuracy and minimal computational complexity in activity recognition [9]. Then exercise rules would be extracted based on the results of exercise classification. Finally, the improper movement is identified by exercise rules we defined in Table 1. As shown in Figure 7, it's the shank angle variation graph during the SLR exercise, including “standard movement” and “raising angle not approximately 45 degree”.

**Figure 8.**
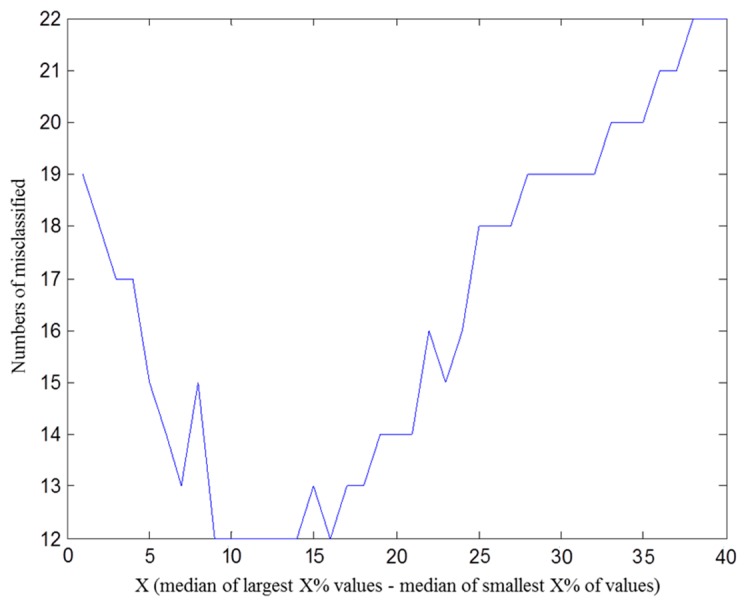
Angle information for improper movement detection.

The calculated angles may contain deviations because sensors are placed on the human body instead of a flat platform. In order to calculate the variation of angles more accurately and compensate for the deviations caused by noises and initial sensor position angles, we calculate the difference between the medians of a fixed proportion of the largest and smallest values, and use this difference as an indicator of angle variation. In other words, we obtain the best angle variation by subtraction between the median of X% largest value and the median of X% smallest value on each time window. Figure 8 is the result that we use some subjects' angle information to determine the X value to get the best accuracy of improper movement detection.

Using angle information to identify improper rehabilitation movements is more intuitive. It is flexible when it comes to modify exercising rules. In order to avoid signal deviation and artificial error, some subjects' angle information is used to define error-tolerance for each exercise, as shown in Table 2.

## 4. Results and Discussion

**Figure 9.**
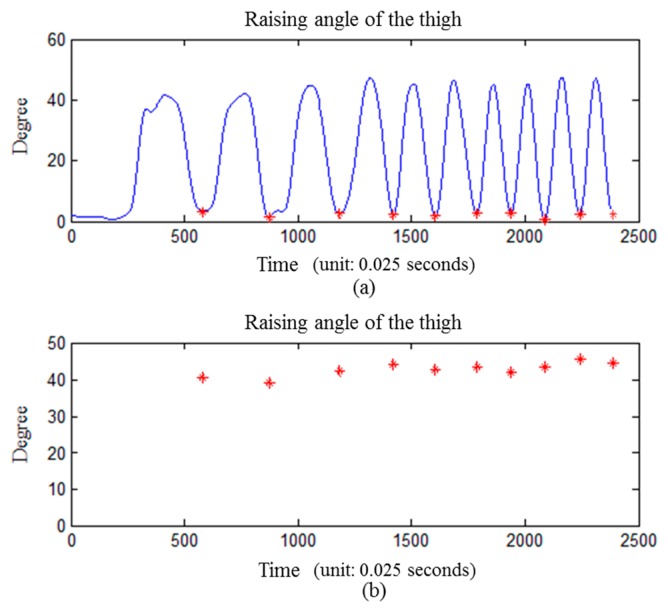
Angle verification with goniometer (thigh raised at 45 degree). (**a**) the raising angle of the thigh while asked to perform a standard movement of SLR for ten times; (**b**) the angle information is obtained by subtraction between median of 10% largest value and median of 10% smallest value on each time window.

Goniometers are widely used in clinical applications. In this study, a goniometer is used to assist subjects in achieving the desired posture in the experiment. For example, we perform an experiment to illustrate the calculated angle which was accomplished by an experienced orthopedic surgeon who measured the angle between the anterior thigh and anterior shank with the goniometer. A subject is asked to perform a standard movement of SLR for ten times with the goniometer properly applied. The angle variation is obtained by subtraction between the median of the 10% largest value and median of 10% smallest value on each time window. The result shows that only one of the movements exceed 45° ± 5°, and none of the movements exceeded 45° ± 10° (10° is the threshold value, as shown in Table 2), as shown in Figure 9.

**Figure 10.**
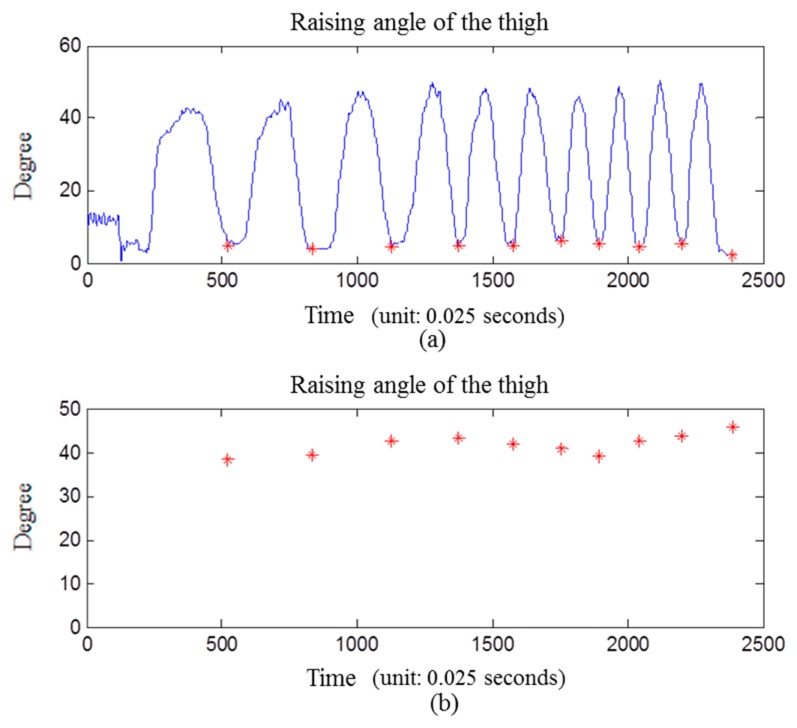
Another method proposed by Takeda [33] to calculate inclination angles. (**a**) the raising angle of the thigh while asked to perform a standard movement of SLR for ten times; (**b**) the angle information is obtained by subtraction between the median of the 10% largest value and median of the 10% smallest value in each time window.

We use another method based on Takeda *et al.* [33] to make a comparison with our angle calculation. The same data are used to compare the two calculation methods. The results show that three of the movements exceed 45° ± 5°, and none of the movements exceeded 45° ± 10°, as shown in Figure 10.

**Figure 11.**
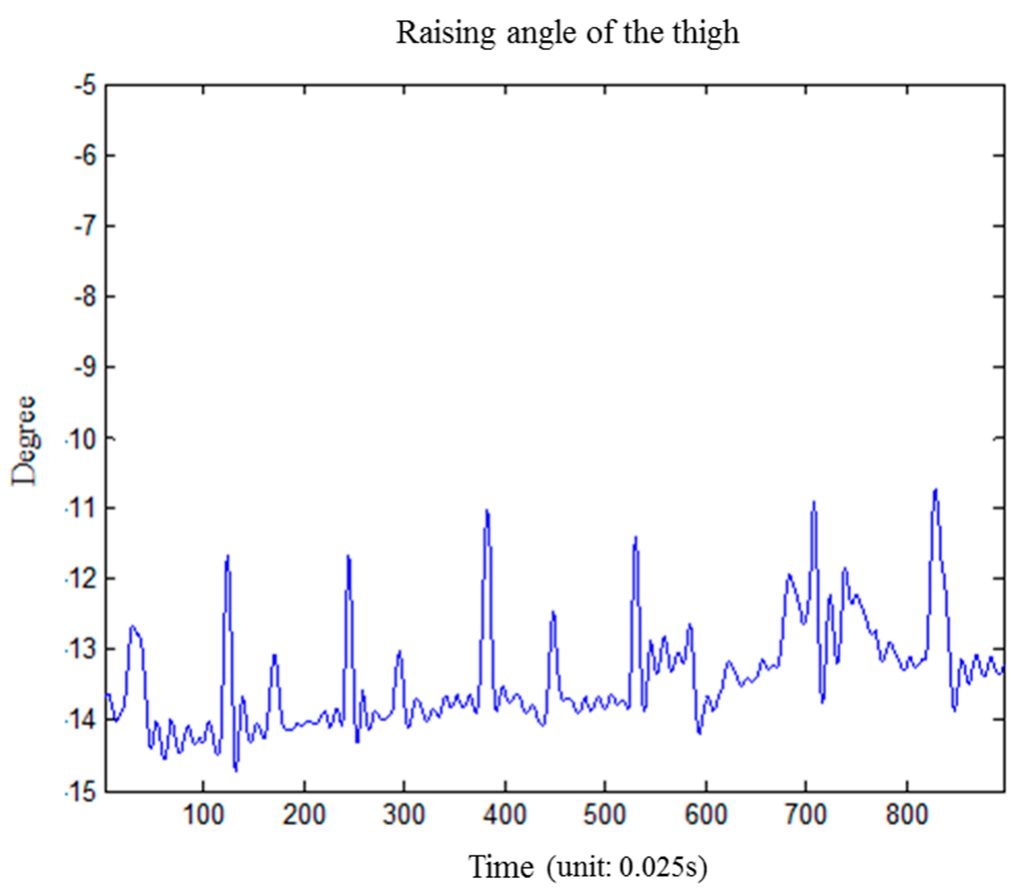
The effect of muscular activity for raising angle of the thigh.

We performed an experiment to investigate the effect of muscular activity on the calculation of thigh raising angle during rehabilitation exercises. We performed seven repetitions of isometric quadriceps exercises and calculated the angle variation of the thigh. The results show that all the variationds were within 3 degree, as shown in Figure 11.

The wearing position of the sensor is a key factor in angle calculation. The sensors are all set at fixed positions in our experimental design, but there must be some deviation in the sensor position. We have performed 40 repetitions of SLR with two different positions of the thigh sensor. One is on anterior surface of the thigh, the same as in our experimental design, and the other is on the lateral surface of the thigh. The differences between the average angles in two different settings were 1.3° for raising angle, 1.0° for joint angle, and 7.6° for external rotation angle. The difference of external rotation angle is larger than others, so it shows that the position of the sensor really has an impact on the angle calculation.

**Figure 12.**
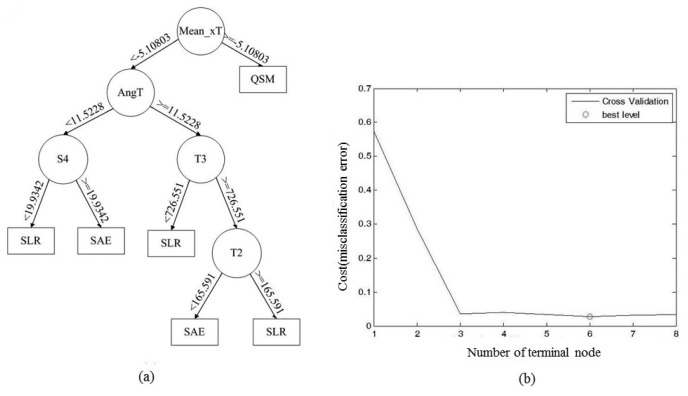
(**a**) Decision tree; (**b**) Number of terminal nodes and CV error curve diagram.

**Figure 13.**
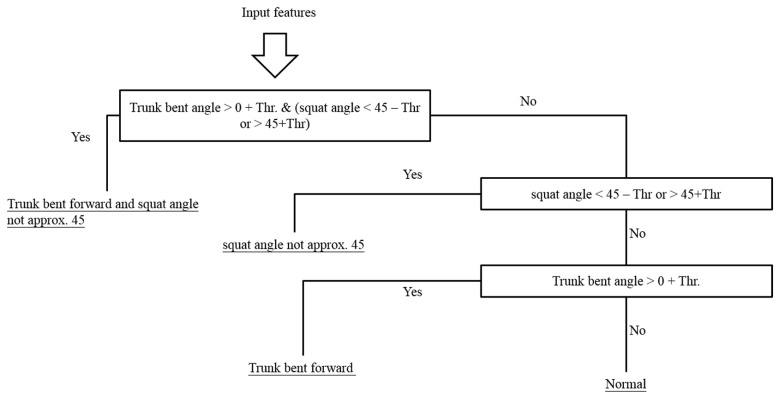
Hierarchical method for QSM.

The generated decision tree is shown in Figure 12a. “Mean_xT” represents the mean value of the thigh sensor accelerometry on the x axis; “AngT” represents the highest thigh raising angle of each movement; “S4” represent the fourth component of FFT on the shank; “T2” and “T3” are the second and third component of FFT of the accelerometer attached on thigh. According to the training results of the decision tree, we can perceive that “Mean_xT” is the most discriminating feature since QSM is a standing movement; and “AngT” is the second most discriminating feature because the biggest difference between SLR and SAE is the thigh-raising angle. The time domain feature, frequency domain feature and angle information can be used as discriminating features of the decision tree in the exercise classification algorithm. We use hierarchical method to identify the improper rehabilitation exercises based on exercise rules. For example, the hierarchical method for QSM is shown in Figure 13.

Ten subjects participated in our experiment. We used 10-fold cross-validation (CV), within-subject CV and leave-one-subject-out CV as methods to confirm the proposed rehabilitation exercise assessment system. 10-fold CV partitions data into 10 subsets, nine for training and one for validating, and 10 rounds of cross-validation are performed using different partitions. Validation results are averaged over all rounds. For within-subject CV, a portion of the samples for a specific subject is used for training, while the remaining samples of the same subject is used for validation. This process is then repeated, each time using a different portion of the subject samples for validation. The overall accuracy is determined by the average of all the cycles for all available subjects. In leave-one-subject-out CV, only one subject is used for validation and the remaining ones are used for training.

The exercise type classification accuracy derived from the three CV methods were 99.29% ± 1.06%, 95.29% ± 8.31%, and 96.86% ± 9.51%, as shown in Tables 3, 4 and 5. The error would be minimized while the tree has six terminal nodes. The accuracy of improper exercise detection achieves 90.14% ± 8.70%, 88% ± 10.62%, and 88% ± 12.03%. The improper identification accuracy considers the misclassified exercise types so that the improper identification accuracy is lower than the exercise type classification accuracy. This result will be affected by the accuracy of exercise type classification and the artificial errors that occurred while the user couldn't meet the movement requirements.

**Figure 14.**
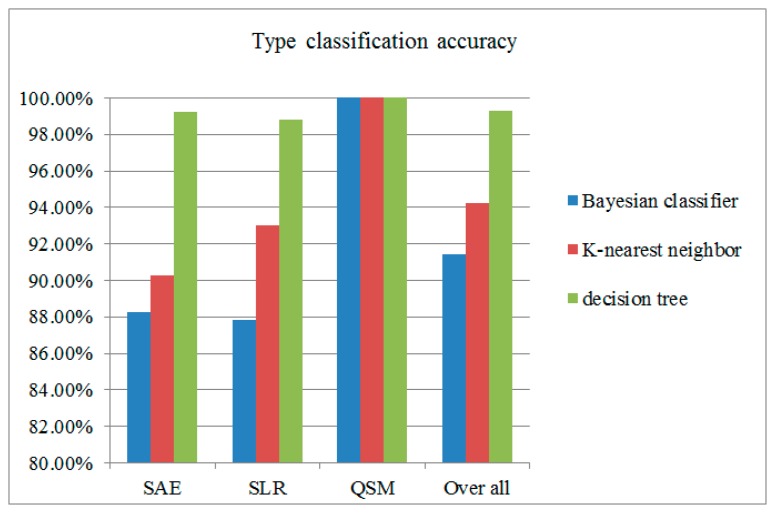
Comparison among classifiers.

We also implement a Bayesian classifier and K-nearest neighbor classifier to compare with decision tree for exercise type classification. 10-fold CV is used to measure the accuracy of those classifiers. The result is shown in Figure 14. The classification results shows that the decision tree has the best accuracy compared with other classifiers. The impact might indicate that some differences between these classifier. The Bayesian and KNN classifier need a large dataset to train the recognition model. The decision tree classified by examining the discriminatory ability of the features one at a time to create a set of rules, which make decision tree classifier outperform others.

## 5. Conclusions

In order to enable the physician and knee OA patients to manage the rehabilitation progress, we have developed a system that can identify the type of exercise movement the user performed and detect deviations from the correct exercise movement, which can allows knee OA patients to take the full benefit of rehabilitation exercises. This system use three wearable accelerometers as signal source, and extracts the signals' time domain feature, frequency domain feature and angle information to identify the type of exercise movement. In the improper identification stage, we use angle information to detect improper rehabilitation movements that can avoid the possible damage caused by inappropriate stress on the injured part. The experimental results have demonstrated that the proposed method provides high exercise type classification and improper movement detection accuracy. It fulfills the requirements of rehabilitation exercise assessment systems. In the setting of home-based rehabilitation for knee OA patients, this system can provide the physician the ability to telemonitor the accuracy of rehabilitation the patient performed, and also provide the patient the ability to self-evaluate whether his/her rehabilitation behavior is correct or not.

In our future work, we will including not only healthy subjects but also knee OA patients of different stages to confirm the proposed mechanism in a clinical situation. Furthermore, we will investigate how the wearing positions of the sensors affect the system performance and the issues on privacy and its invasion.

## Figures and Tables

**Table 1.** Alternations of three rehabilitation exercises.

**Rehabilitation Exercise**	**Label**	**Times**
Short-Arc Exercise (SAE)	1. Normal	10
	2. Initial knee flexion angle >25°	10
	3. Knee not fully extended	10
	4. Both 2 and 3	10

Straight Leg Raise (SLR)	1. Normal	10
	2. Knee not fully extended	10
	3. Hip joint external rotation	10
	4. Raise angle not approx. 45°	20
	5. Both 2 and 3	10

Quadriceps	1. Normal	10
Strengthening	2. Trunk bent forward	10
Mini-squats	3. Knee angle not approx. 45°	10
(QSM)	4. Both 2 and 3	10

Total		140

**Table 2.** Error tolerance threshold (angle).

**Angle Parameter**	**Threshold (°)**
Shank terminal angle (SAE)	7
Knee joint initial flexion angle (SAE)	7
Thigh raising angle (SLR)	10
Trunk bent forward angle (QSM)	15
Squat angle (QSM)	15
Knee flexion angle (SLR)	20
Hip external rotation angle (SLR)	20

**Table 3.** 10-fold cross-validation.

**Exercise**	**Type Classification Accuracy**	**Improper Identification Accuracy**
SAE	99.25% ± 1.05%	93.00% ± 8.34%
SLR	98.83% ± 1.58%	86.17% ± 9.03%
QSM	99.79% ± 0.04%	93.25% ± 8.70%
All	99.29% ± 1.16%	90.14% ± 8.70%

**Table 4.** Within-subject cross-validation.

**Exercise**	**Type Classification Accuracy**	**Improper Identification Accuracy**
SAE	97.75% ± 3.22%	82.00% ± 12.19%
SLR	91.17% ± 12.67%	85.17% ± 9.46%
QSM	98.25% ± 4.09%	92.00% ± 8.70%
All	95.72% ± 8.31%	86.39.00% ± 10.62%

**Table 5.** Leave-one-subject-out cross-validation.

**Exercise**	**Type Classification Accuracy**	**Improper Identification Accuracy**
SAE	92.75% ± 15.57%	86.25% ± 16.12%
SLR	97.83% ± 4.31%	86.50% ± 9.24%
QSM	100% ± 0%	92.00% ± 8.70%
All	96.86% ± 9.51%	88.25% ± 12.03%
